# Toxicity Study and Antibacterial Effects of the Leaves Extracts of *Boscia coriacea* and *Uvaria leptocladon*

**DOI:** 10.4314/ejhs.v32i4.20

**Published:** 2022-07

**Authors:** Sintayehu Tsegaye Tseha, Yalemtsehay Mekonnen, Asnake Desalegn, Amelework Eyado, Melaku Wondafarsh

**Affiliations:** 1 Department of Microbial, Cellular and Molecular Biology, College of Natural and Computational Sciences, Addis Ababa University, Ethiopia; 2 Department of Biology, College of Natural and Computational Sciences, Arba Minch University, Ethiopia; 3 Department of Zoological Sciences, College of Natural and Computational Sciences, Addis Ababa University, Addis Ababa, Ethiopia; 4 Department of Microbial, Cellular and Molecular Biology, College of Natural and Computational Sciences, Addis Ababa University, Addis Ababa, Ethiopia; 5 Department of Zoological Sciences, College of Natural and Computational Sciences, Addis Ababa University, Addis Ababa, Ethiopia; 6 Department of Plant Biology and Biodiversity Management, College of Natural and Computational Sciences, Addis Ababa University, Addis Ababa, Ethiopia

**Keywords:** Boscia coriacea, Uvaria leptocladon, antibacterial activity, toxicity, phytochemicals, antibiotics

## Abstract

**Background:**

The objective of this study was to evaluate the in vivo toxicity and antibacterial activity of the leaves extracts of Boscia coriacea and Uvaria leptocladon.

**Methods:**

Extraction was performed using 80% methanol by maceration and Soxhlet extraction method. Evaluation of the acute toxicity of the extracts was based on the Organization for Economic Cooperation and Development (OECD) guideline. Evaluation of antibacterial activity of the extracts was done by agar well diffusion assay. Determinations of minimum inhibitory concentrations (MIC) of the extracts were performed by broth macro-dilution method. The checkerboard method was used for the determination of combined effect of antibiotics and the extracts. Paired T-test and one way analysis of variance were used for statistical analysis.

**Results:**

B. coriacea and U. leptocladon have no toxic effect in Swiss albino mice up to dose of 5000 mg/kg. B. coriacea and U. leptocladon showed antibacterial activity at concentration of 500 mg/ml. The chloroform-methanol fraction of B. coriacea and U. leptocladon showed the highest antibacterial activity at concentration of 25 mg/ml. The MIC and minimum bactericidal concentration (MBC) of B. coriacea were 125 mg/ml and 250 mg/ml, respectively. The MIC of U. leptocladon ranged from 31.25 mg/ml to 62.5 mg/ml, while its MBC ranged from 62.5 to 125 mg/ml. The combination assay of B. coriacea and the antibiotics showed additive effect, while U. leptocladon and the antibiotics showed indifferent effect.

**Conclusion:**

The findings showed that U. leptocladon and B. coriacea leaves extracts have antibacterial activity and no toxicity in animal model.

## Introduction

Medicinal plants are major sources of drug development. In recent years, antimicrobial drugs resistance has become one of the major challenges of controlling infectious diseases. Antimicrobial drugs resistance is estimated to account for 700,000 deaths every year in the world (1).

Pathogenic bacteria such as *Salmonella typhi, Staphylococcus aureus, Escherichia coli, Klebisella pneumoniae* and *Pseudomonas aeruginosa* have already developed resistance against commonly used antimicrobial drugs (2–4). The most important interventions to combat antimicrobial drugs resistance is to search for effective newer antimicrobial drugs.

Plants that are used in herbal traditional medicine can offer a promising source of chemicals with antibacterial activities (5–12). Different *Boscia* and *Uvaria* species have been shown to have antibacterial activities against various pathogenic bacteria species (5, 6, 9–12). The genus *Uvaria* belongs to the family Annonaceae that is widely distributed in Ethiopia. For example *Uvaria leptocladon* is found in Gamo Gofa, Kefa, Konso (Alie), Sidamo and regions of Ethiopia (13). The genus *Boscia* belongs to the family Capparidaceae, which is also widely distributed in Ethiopia. For instance *Boscia coriacea* is found in Kefa, Gamo Gofa, Sidamo, Konso, Bale and Hararge regions of Ethiopia (14). Therefore, based on the traditional use, *Boscia coriacea* and *Uvaria leptocladon* may have some antibacterial activities. Hence, the aim of this study was to evaluate the acute toxicity in animal model and antibacterial activity of the leaves extracts of *B. coriacea* and *U. leptocladon* on some pathogenic bacteria.

## Materials and Methods

**Plant collection**: Fresh leaves of *U. leptocladon* and *B. coriacea* were collected in April 2021 from Alie and Konso, located in Southern Nation, Nationalities and People Region, Ethiopia (SNNPR). After the plant materials were authenticated by one of the authors, a Botanist at the Department of Plant Biology and Biodiversity Management, a voucher specimen of each plant (ST001 and ST002, representing *B.coriacea* and *U. leptocladon*, respectively) was deposited at the National Herbarium, College of Natural and Computational Sciences, Addis Ababa University. Then, the leaves samples were cleaned and allowed to dry in shade avoiding direct sun light.

**Extraction**: The powdered leaves of *B.coriacea* (1 kg) and *U. leptocladon* (1 kg) were extracted by maceration and Soxhlet extraction method using 80% methanol (MeOH). The powdered leaves of each plant species (1 kg) was extracted by 10 liters of solvent (80% MeOH)). Then, the mixture was filtered using Whatmann no.1 filter paper. The MeOH was removed from the filtrate by evaporation using rotary evaporator. The water component of the mixture was removed by lyophilization using a lyophilizer equipment (Christ, ALPHA 2-4-LD Plus). The 80% MeOH extract was fractionated using chloroform (CHCl_3_), MeOH and CHCl_3_-MeOH (1:1) in the Department of Chemistry, College of Natural and Computational Sciences, Addis Ababa University. The extraction yield (w/w) was 5% and 18% for maceration and soxhlet extracts, respectively. All the extracts were stored at -20°C until experiments were conducted.

**Acute oral toxicity study**: The oral acute toxicity test was done based on the Organization for Economic Cooperation and Development guideline (OECD) for toxicity study (15). The toxicity experiment was performed in female Swiss albino mice that weighed in the range of 23–31g and aged from 6 to 8 weeks. After starving the mice for three hours, a single dose of the 80% MeOH extract dissolved in normal saline was given orally. After dividing the mice randomly into five groups and each group containing five mice, the mice were then given 1000, 1500, 2000 and 5000 mg/kg of the extract. The mice in the control group were administered normal saline. The mice were monitored for any behavioral and physical changes as well as mortality continuously for 14 days. Body weight of the mice was measured on day zero before administration of the extract and on days 7 and 14 after administration of the extract.

For determination of change in packed cell volume (PCV) of Swiss albino Mice, PCV was measured at day zero and day 14 by taking blood sample from the tips of tails of the mice. Heparinized capillary tubes were used for collection of blood from the tail of each mouse.

The capillary tubes were filled with blood up to three-fourth of their volume and sealed at the dry end with sealing clay. The tubes were then placed in a micro-haematocrit centrifuge and centrifuged for 5 min at 12,000 rpm. Finally, the tubes were taken out of the centrifuge to calculate PCV.

PCV was calculated by the following formula:-


$$\begin{array}{c} \text{PCV=}\underline{\text{Vol. of total erythrocytes in a given vol. of blood}}\text{ X 100}\\ \text{Total blood volume} \end{array}$$

Note: Vol. stands for volume.

**Antibacterial Activity Test**

**Test organisms**: The pathogenic bacteria used in this study were *E. coli* (ATCC25922), *L. monocytogenes* (ATCC19115), *P. aeruginosa* (ATCC27853), *S. typhimurium* (ATCC14028); *K. pneumoniae* (ATCC700603) and *S. aureus* (ATCC25923). All the bacteria were obtained from the Ethiopian Public health institute (EPHI), Addis Ababa, Ethiopia.

**Inoculum preparation**: Pure colony of each bacterium species was allowed to grow in nutrient broth at 37 °C for 24 hrs. Then, each colony of bacterium was transferred to nutrient agar and allowed to grow at 37 °C for 24 hrs. Inoculums were standardized by comparing with 0.5 McFarland turbidity standards (16).

**Reference drugs**: Amoxicillin (25 µg/ml) was used as standard drug against *S. aureus* and *L. monocytogenes.* Whereas, ciprofloxacin (5 µg/ml) was used as reference drug against *E. coli, S. typhimurium, K. pneumoniae* and *P. aeruginosa* (17). Both the amoxicillin and the ciprofloxacin were obtained from the Ethiopian Pharmaceuticals Manufacturing Company SC (EPHARM), Addis Ababa, Ethiopia.

**Agar well diffusion assay**: The antibacterial activity test of the plant extracts was done using agar well diffusion assay (18). The test bacteria (100 µl) were inoculated on Petri dishes that contain solidified Mueller Hinton agar (MHA) media using sterile cotton swab.

Then, wells that are 6 mm in diameter were prepared in the MHA medium using a sterile Cork borer. 50 µl of 80% methanol extract (500 mg/ml) that was dissolved in 10% Dimethyl sulfoxide (DMSO) was dispensed into the wells. In addition, 50 µl of the positive and negative controls were also loaded in the agar wells. DMSO (10%) was used as a negative control. Then, the plates were incubated at 37ºC for 24 hours. Diameter of the zone of inhibition (in millimeter) was measured to determine the sensitivity of the test bacteria to the extracts and the given drugs using Vernier caliper. Each test was performed in triplicate.

**Determination of minimum inhibitory concentration (MIC) and minimum bactericidal concentration (MBC)**: Two-fold broth macro-dilution was used for the determination of the minimum inhibitory concentration (MIC). First, each test organism (100 µl) was added to test tubes that contain 5 ml of Mueller Hinton Broth. Then, 100 µl of diluted 80% MeOH extract of the extract obtained separately from *U. leptocladon* and *B.coriacea* were added to the test tubes. Following this, the mixtures in the test tubes were incubated at 37°C for 24 hrs. For non-contamination/sterility control, the 80% MeOH extract without bacteria was also incubated at 37°C for 24 hrs. Growth control was tested by allowing 100 µl of each bacterium species to grow in Mueller Hinton Broth that did not contain the extracts and Triphenyl tetrazolium chloride (TTC) was used as growth indicator. The MIC refers to the lowest concentration of an extract that brings about absence of visible growth of bacteria (19).

After determining the MIC of the extracts, the minimum bactericidal concentrations (MBC) of the extracts were determined by sub-culturing the broth without visible growth on MHA at 37°C for 24 hours. The MBC was defined as the lowest concentration of the sub-cultured plates which did not show any visible growth of bacteria (19). The tests were performed in triplicate.

**Combined effect of 80% methanol leaves extracts of *U. leptocladon* and *B. coriacea* and antibiotics**: The combined effect of the extracts and the reference drugs was evaluated by using checker board micro-dilution method with modification (20). Two fold serial dilutions of each plant extract and the reference drugs were performed to yield the following concentrations of the extracts and the antibiotics: ciprofloxacin (2.5 to 0.3125 µg/ml); amoxicillin (12.5 to 1.56 µg/ml) and the extracts (250 mg/ml to 31.25 mg/ml). Five ml of nutrient broth was dispensed into each test tube. Following this, 100 µl of each test organism was added to their respective test tubes. 50 µl of each dilution of the extract and each drug dilution were added to the test tubes. 20 µl of TTC solution was added into each test tube. The test tubes were incubated at 37°C for 24 hrs. The lowest concentration of the combination of the extract and antibiotics in which the colorless TTC did not appear deep red (pink) was taken as MIC for the combination of the extract and antibiotics. Growth control and sterility control was performed for each experiment.

The fractional inhibitory concentration index (FICI) was calculated to determine the interaction between the extract and the reference drugs by using the following formula:

$$\begin{array}{ccc} \text{FICI=}\underline{\text{MICa in comb}} & \text{+} & \underline{\text{MICb in comb}}\\ \text{MICa} & & \text{MICb} \end{array}$$

Where, MICa is MIC of plant extract and MICb is MIC of antibiotics. Comb stands for combination. Interpretation of the FICI was as follows: FICI > 4 is antagonism; FICI >1–4 is indifference; FICI > 0.5–1 is additive and FICI ≤ 0.5 is synergy (21).

**Ethical consideration**: Ethical clearance was obtained from the Institution Review Board of the College of Natural and Computational Sciences of Addis Ababa University. Copy is attached as additional file.

**Data analysis**: One way analysis of variance (ANOVA) was used for analysis of differences between bacterial growth inhibition caused by the leaves extracts of the plants and controls. Paired sample t-test was used for analysis of the differences between body weight and PCV of Swiss albino mice before and after administration of the extracts. P-value < 0.05 was considered statistically significant.

## Results

**Acute toxicity of leaves extracts of *U. leptocladon and B. coriacea***: The oral acute toxicity study revealed that the 80% methanol leaves extracts of *U. leptocladon* and *B. coriacea* have no toxic effect except the erection of hair that was observed for a few minutes after administration of the extracts.

No mouse died throughout the 14-days follow up period, suggesting that median lethal dose of the leaves extracts of *U. leptocladon* and *B. coriacea* is greater than 5000 mg. There was no statistically significant difference between the mean PCV and body weight of the Swiss albino mice before and after administration of 80% methanol leaves extracts of *B. coriacea* and *U. leptocladon*.

**Antibacterial effects of the leaves extracts of *U. leptocladon* and *B. coriacea***: Table 1 shows the diameter of zone of growth inhibition caused by 80% methanol leaves extracts of *B. coriacea* and *U. leptocladon* against the tested bacteria species. The 80% methanol leaf extract of *U. leptocladon* inhibited the growth of *P. aeruginosa, S. aureus, E. coli, S. typhimurium* and *L.monocytogenes*. Whereas, the leaf extract of *U. leptocladon* did not result in growth inhibition against *K. pneumoniae*. The leaf extract of *B. coriacea* showed growth inhibition only against *P. aeruginosa, S. aureus* and *L. monocytogene*s. The CHCl_3_-MeOH (1:1) fraction of *U. leptocladon* and *B. coriacea* leaves extracts showed the highest antibacterial activity against all tested bacteria species (Table 2 and 3). There were statistically significant differences (P<0.05) between the mean diameter of zone of growth inhibitions of the reference drugs and the leaves extracts of *U. leptocladon* and *B. coriacea.*

**Table 1:** Growth inhibition of 80% methanol leaves extracts of *U. leptocladon* and *B.coriacea* against different bacteria species at concentration of 500 mg/ml

Bacteria species	Diameter of zone of growth inhibition in millimeter				
	
	*B.coriacea*	PI of *B.coriacea*	*U.leptocladon*	PI of *U.leptocladon*	Positive control
*S. typhimurium*	0±0	0%	20±0.6	54.1%	37±0.6
*K. pneumonia*	0±0	0%	0±0	0%	32±0.6
*L. monocytogenes*	21±0.6	60%	21±0.6	60%	35±1.6
*E. coli*	0±0	0%	20±1.6	66.7%	30±0.6
*S. aureus*	21±0.6	52.5%	20±1.6	50%	40±0.6
*P. aeruginosa*	20±1.6	57.1%	18±0.6	51.4%	35±0.6

*Note: The positive controls are ciprofloxacin and amoxicillin. Ciprofloxacin (5 µg/ml) was used as positive control against *E. coli, S. typhimurium, K. pneumoniae* and *P. aeruginosa*. Whereas, amoxicillin (25 µg/ml) as used as positive control against *S. aureus* and *L. monocytogenes*. PI indicates percent inhibition of the leaves extracts of *U. leptocladon* and *B. coriacea* in reference with the standard antibiotics*

**Table 2:** Growth inhibition of leaf fractions of *B. coriacea* against different bacteria species at concentration of 25 mg/ml

Bacteria species	CHCl_3_	PI of CHCl_3_	CHCl3:MeOH	PI of CHCl3:MeOH	MeOH	PI of MeOH	Positive control
*S. aureus*	0 ±0	0%	20 ±1.6	50%	0 ±0	0%	40±1.6
*P. aeruginosa*	0 ±0	0%	16 ±0.6	45.7%	0 ±0	0%	35 ±0.6
*L. monocytogenes*	0±0	0%	10±1.6	28.6%	8±0.6	22.8%	35±0.6

Note: The positive controls are ciprofloxacin and amoxicillin. Ciprofloxacin (5 µg/ml) was used as positive control against *P. aeruginosa*. Whereas, amoxicillin (25 µg/ml) as used as positive control against *S. aureus* and *L. monocytogenes*. PI indicates percent inhibition of fractions of the leaf extract of *B. coriacea* in reference with the standard antibiotics.

**Table 3:** Growth inhibition of leaf fractions of *U. leptocladon* against different bacteria species at concentration of 25 mg/ml.

Bacteria species	CHCl_3_	PI of CHCl_3_	CHCl_3_:MeOH	PI of CHCl_3_:MeOH	MeOH	PI of MeOH	Positive control
*S. aureus*	0 ±0	0%	12 ±1.6	30%	0±0	0%	40±0.6
*P. aeruginsa*	0 ±0	0%	15 ±1.6	42.9%	0 ±0	0%	35 ±0.6
*L. monocytogenes*	8 ±0.6	22.8%	15±1.6	42.9%	10±1.6	28.6%	35±0.6
*E. coli*	8±0.6	26.7%	16±0.6	53.3%	8±0.6	26.7%	30±1.6
*S. typhimurium*	0±0	0%	12±0.6	32.4%	0±0	0%	37±0.6

Note: The positive controls are ciprofloxacin and amoxicillin. Ciprofloxacin (5 µg/ml) was used as positive control against *E. coli, S. typhimurium*, and *P. aeruginosa*. Whereas, amoxicillin (25 µg/ml) as used as positive control against *S. aureus* and *L. monocytogenes*. PI indicates percent inhibition of fractions of the leaf extract of *U. leptocladon* in reference with the standard antibiotics.

The percent inhibition of the 80% methanol leaf extract of *B. coriacea* ranges from 0% to 60% (Table 1). Whereas, the percentage inhibition of the 80% methanol leaf extract of *U. leptocladon* ranges from 0% to 66.7%. The CHCl_3_: MeOH fraction of both *B. coriacea and U. leptocladon* has the highest % inhibition (Tables 2 and 3).

**MIC and MBC of the leaves extracts of *U. leptocladon* and *B. coriacea***: The MIC and MBC of *B. coriacea* against *L. monocytogenes, S. aureus* and *P. aeruginosa* were demonstrated to be 125 mg/ml and 250 mg/ml, respectively. The MIC of *U. leptocladon* was 62.5 mg/ml against *E.coli, S. typhimurium*, and *L. monocytogenes* and 31.25 mg/ml against *S.aureus* and *P. aeruginosa*. The MBC of *U. leptocladon* was 125 mg/ml against *E.coli, S. typhimurium*, and *L. monocytogenes* and 62.5 mg/ml against *S. aureus* and *P. aeruginosa* (Table 4).

**Table 4:** MIC and MBC of the leaves extracts of *U. leptocladon* and *B. coriacea* against different bacteria species

Plant species	*L.monocytogenes*		*S. aureus*		*P. aeruginosa*		*S. typhimurium*		*E. coli*	
	
	MIC	MBC	MIC	MBC	MIC	MBC	MIC	MBC	MIC	MBC
*B. coriaca*	125	250	125	250	125mg	250	Not	Not	Not	Not
	mg/ml	mg/ml	mg/ml	mg/ml	/ml	mg/ml	tested	tested	testes	tested
*U.*	62.5	125	31.25	62.5	31.25m	62.5	62.5	125	62.5	125
*leptocladon*	mg/ml	mg/ml	mg/ml	mg/ml	g/ml	mg/ml	mg/ml	mg/ml	mg/ml	mg/ml

**Combined effects of antibiotics and the leaves extracts of *U.leptocladon* and *B. coriacea***: The study proved that the combination of *B. coriacea* crude leaf extract and the standard antibiotics have an additive effect against *P. aeruginosa, L. monocytogenes* and *S. auerus*. Whereas, the combination of *U. leptocladon* and the standard antibiotics showed indifferent effect against *P. aeruginosa, L. monocytogenes, S. auerus, E. coli* and *S. typhimurium* (Tables 5 and 6).

**Table 5:** MIC of combination of antibiotics and leaf extract of *B. coriacea* against different bacteria species

Bacteria species	MIC of *B.* *coriacea*	MIC of antibiotics	MIC of *B.* *coriacea* in comb	MIC of antibiotics in comb	FICI	Interpretation of FICI
*S. aureus*	125 mg/ml	12.5 µg/ml	62.5 mg/ml	3.125µg/ml	0.75	Additive
*P. aeruginosa*	125 mg/ml	2.5 µg/ml	62.5 mg/ml	0.625µg/ml	0.75	Additive
*L. monocytogenes*	125 mg/ml	12.5µg/ml	62.5 mg/ml	3.125 µg/ml	0.75	Additive

Note: The antibiotics are ciprofloxacin and amoxicillin. Ciprofloxacin (5 µg/ml) was used in combination with the leaf extract of *B. coriacea* against *P. aeruginosa*. Whereas, amoxicillin (25 µg/ml) was used in combination with the leaf extract of *B. coriacea* against *S. aureus* and *L. monocytogenes*. FICI > 4 is antagonism; FICI >1–4 is indifference; FICI > 0.5–1 is additive and FICI ≤ 0.5 is synergy. The formula to calculate the FICI was described in the materials and method section of the manuscript. Comb indicates combination.

**Table 6:** MIC of combination of antibiotics and leaf extract of *U. leptocladon* against different bacteria species

Bacteria species	MIC of *U.leptocladon*	MIC of antibiotics	MIC of antibiotics in comb	MIC of *U.leptocladon* in comb	FICI	Interpretation of FICI
*E. coli*	62.5 mg/ml	0.625µg/ml	0.625 µg/ml	62.5 mg/ml	2	Indifference
*S. typhimurium*	62.5 mg/ml	1.25µg/ml	0.625 µg/ml	62.5 mg/ml	1.5	Indifference
*L. monocytogenes*	62.5 mg/ml	12.5 µg/ml	6.25 µg/ml	62.5 mg/ml	1.5	Indifference
*S. aureus*	31.25 mg/ml	12.5 µg/ml	3.125 µg/ml	31.25 mg/ml	1.25	Indifference
*P. aeruginosa*	31.25 mg/ml	2.5 µg/ml	0.625 µg/ml	62.5 mg/ml	2.25	Indifference

Note: The antibiotics are ciprofloxacin and amoxicillin. Ciprofloxacin (5 µg/ml) was used in combination with the leave extracts against *E.coli, S. typhimurium*, and *P. aeruginosa*. Whereas, amoxicillin (25 µg/ml) was used in combination with the leaf extract against *S. aureus* and *L. monocytogenes*. FICI > 4 is antagonism; FICI >1–4 is indifference; FICI > 0.5–1 is additive and FICI ≤ 0.5 is synergy. The formula to calculate the FICI was described in the materials and method section of the manuscript. Comb indicates combination.

## Discussion

The results of the present study have shown that the 80% methanol leaves extracts of *B. corieacea* and *U. leptocladon* was found to be safe in the Swiss albino mice tested. The safety profile of *B. coriacea* and *U. leptocladon* leaves extracts noted in this study is comparable with other studies that evaluated toxicology of other *Uvaria* and *Boscia* species (22–24).

The antibacterial activity of the leaf extract of *B. coriacea* against gram positive and gram negative bacteria observed in this study is in line with reports that have been made by other researchers which studied antibacterial activity of other *Boscia* species leaf extract such as *B. albitrunca* (25). However, the insensitivity of *E. coli* and *K. pneumoniae* to *B. coriacea* leaf extract observed in the present study is different with a finding of another study that showed antibacterial activity of *Boscia* species against *E. coli* and *K. pneumoniae* (25). Previous works have shown the presence of alkaloids, flavonoids and terpenoids in the leaves extracts of *Boscia* species (6, 25).

The antibacterial activity of *U. leptocladon* that was observed in this study corroborate the findings of previous study that reported antibacterial activity of the leaf extract of other *Uvaria* species against gram positive and gram negative bacteria (26). The antibacterial activity of *U. leptocladon* can be explained by the fact that *Uvaria* species leaves extracts contain phytochemicals that have potent antimicrobial activities, including flavonoids and alkaloids (20, 25). The highest antibacterial activity of CHCl_3_: MeOH (1:1) fraction of the two plants leaves extracts indicates that the antimicrobial constituents are contained in these fractions.

The percentage inhibition of *B.coracea* (up to 60%) and *U. leptocladon* (up to 66.7%) documented in this study indicates the potent antibacterial properties of *B. coracea a*nd *U. leptocladon* leaves extracts against some gram positive and gram negative bacteria. Worth noting is the 80% MeOH extract of *B. coracea* resulted in 60% inhibition of *L. monocytogenes* and 57.1% inhibition of *P. aeruginosa* as compared to the standard drugs and the 80% MeOH extract of *U. leptocladon* resulted in 60% inhibition of *L. monocytogenes* and 66.7% inhibition of *E.coli* as compared to the standard drugs, demonstrating the potential of these extracts for further antibacterial study (Table 1). Furthermore, the CHCl3: MeOH fraction of *B. coracea* has resulted in 45.7% inhibition on *P. aeruginosa* (Table 2), while *U. leptocladon* has shown 53.3% inhibition on *E. coli* (Table 3). These results substantiated the antibacterial effect of these extracts.

Different studies have shown that plant extracts that possess antimicrobial properties may enhance antimicrobial activities of standard drugs when used concurrently with the antibiotics (27–29). The mechanisms of this plant extract-drug interaction include sequential inhibition of common biochemical pathways, enhancing the diffusion of antimicrobials, and inhibition of protective enzymes (30). This is the first study that investigated antibacterial activity of combination of antibiotics and extracts of *Uvaria* and *Boscia* species. An additive effect refers to a joint effect of antimicrobials that is equal to the sum of effects of the individual agents (21). The additive effects of the combination of standard drugs and *B. coriacea* leaf extract against *S.aureus, P. aeruginosa* and *L. monocytogenes* noted in this study suggests the possibility of enhancing therapeutic efficacy of standard drugs by using the antibiotics in combination with *B. coriacea* leaf extract. However, such combination use needs further *in vivo* assessment. In contrast to the *B. coriacea* leaf extract that showed an additive effect when used in combination with the antibiotics, the *U. leptocladon* leaf extract demonstrated indifferent effect when used together with the antibiotics. Indifferent effect is a joint effect of antimicrobials that is equal to the effects of the individual agent (21).

In conclusion, 1. *U. leptocladon* and *B. coriacea* leaves extracts have no toxic effects with median lethal dose of greater than 5000 mg/kg; 2. *U. leptocladon* and *B. coriacea* leaves extracts have antibacterial activities; 3. The combination of leaf extract of *B. coriacea* and the reference drugs have an additive effect against *S. aureus, P. aeruginosa* and *L. monocytogenes*; 4. The combination of the leaf extract of *U. leptocladon* and the reference drugs have indifferent effect against *E. coli, S. typhi, S. aureus, P. aeruginosa* and *L. monocytogenes*. Further study is needed on the efficacy and *in vivo* sub-acute and chronic toxicity of *U. leptocladon* and *B. coriacea* leaves extracts.
